# Reply to the ‘Comment on “Structural, electrical and multiferroic characteristics of lead-free multiferroic: Bi(Co_0.5_Ti_0.5_)O_3_–BiFeO_3_ solid solution”’ by P. E. Tomaszewski, *RSC Adv.*, 2022, **12**, DOI: 10.1039/D1RA08415A

**DOI:** 10.1039/d2ra03468a

**Published:** 2022-11-14

**Authors:** Nitin Kumar, Alok Shukla, Nripesh Kumar, R. N. P. Choudhary, Ajeet Kumar

**Affiliations:** Department of Physics, National Institute of Technology Mizoram Aizawl-796012 India nitinphysicskushawaha@gmail.com; Department of Physics, Siksha O Anusandhan (Deemed to be University) Bhubaneswar-751030 India; School of Physics, University of Hyderabad Hyderabad-500046 India

## Abstract

The cobalt and titanium modified BiFeO_3_ [*i.e.*, Bi(Co_0.40_Ti_0.40_Fe_0.20_)O_3_; referred to as BCTF80/20] solid solution was synthesized *via* a simple and cost effective solid-state technique, and numerous sets of studies (structural, elemental, electrical, leakage current, multiferroic and other properties) were carried out and reported. The basic structural symmetry was investigated and phase identification of the prepared samples was carried out by analyzing powder X-ray diffraction data through the widely used “POWDMULT” software. From the XRD pattern [Fig. 2(a) of *RSC Adv.*, 2018, **8**, 36939], it is clear that almost all the reflection peaks (including those that appear to be split) have been indexed to a single phase (based on the best agreement between experimental and calculated interplanar distances and minimum standard deviation) system using the above software. The lattice parameters, average crystallite size, cell volume, and micro-strain value are strongly affected by the addition of Co and Ti into the bismuth ferrite. The significant enhancement of various parameter (*i.e.*, electrical, multiferroic and so on) values of BCTF80/20 ceramics may make them promising candidates for the development of new generation electronic devices.

## Abstract 2

A solid solution of Bi(Co_0.40_Ti_0.40_Fe_0.20_)O_3_ was synthesized *via* a simple and cost effective solid-state technique, and various properties were investigated. Phase identification of the samples was performed using powder X-ray diffraction data with POWDMULT software.

## Graphical abstract

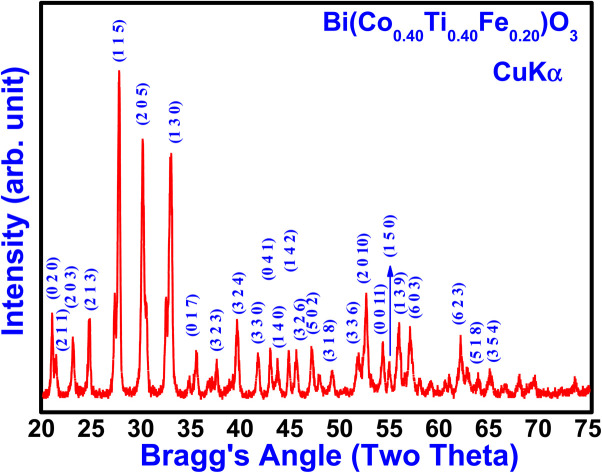

The present manuscript mainly deals with the scientific evidence/description raised by Paweł E. Tomaszewski regarding our previously published work.^1^ The published solid-solution based compound [Co/Ti modified BiFeO_3_, *i.e.*, Bi(Co_0.40_Ti_0.40_Fe_0.20_)O_3_ compound] was prepared *via* a cost effective solid-state reaction technique.^1^ The published work (chemical composition of Bi(Co_0.40_Ti_0.40_Fe_0.20_)O_3_; referred to as BCTF80/20) also provides a detailed description of the synthesis process and the structural, electrical and multiferroic characterization.^1^ The following key points provide an explanation of our response to the comment. For the said chemical composition, the required amounts of high-purity (>99.9% pure) powders of the bismuth trioxide (Bi_2_O_3_), cobalt monoxide (CoO), titanium dioxide (TiO_2_) and iron oxide (Fe_2_O_3_) ingredients were taken (in their stoichiometric amounts) by weighing them using a high-precision digital balance, and mixed thoroughly. These finely ground oxide powders were mixed homogeneously and calcined at the optimal temperature (1030 K for 8 hours). The following chemical reaction was performed to synthesize the Bi(Co_0.40_Ti_0.40_Fe_0.20_)O_3_ compound at ambient pressure under a controlled heating and cooling cycle.
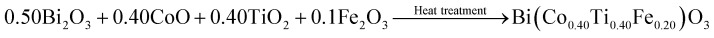


After synthesizing the BCTF80/20 compound, the structural data of the calcined powder were recorded by using a powder X-ray diffractometer. The preliminary structural analysis (crystal symmetry and lattice parameters) was performed with limited X-ray diffraction data through the widely used “POWDMULT” indexing software.^2^ From the XRD pattern in the reported work [Fig. 2(a) in *RSC Adv.*, 2018, **8**, 36939], it is clear that almost all the reflection peaks (including those that appear to be split) have been indexed to a single phase system. However, a bi-phasic/multiple phase nature also cannot be ruled out based on the other program used for this. We have taken the ingredients according to the single-phase, but the commenter’s [Paweł E. Tomaszewski] suggestion of a bi-phasic/multiple phase nature may require other ingredients and chemical compositions. On the basis of the best agreement between the observed and calculated inter-planar spacing of most of the reflections, and minimum/smallest standard deviations (0.0022 Å), the orthorhombic crystal symmetry was selected and presented.^1^

In addition, it should be noted that for structural analysis of ceramic samples and single crystals, nowadays, different advanced level computer software (only) is used because of the complexity of the unknown/known structure of materials. It is well-known that, unlike for single crystals, the complete crystal symmetry/structure of ceramic samples/reported compounds, especially complex ones, cannot be accurately determined with limited powder X-ray diffraction data. It is also known that there is much difference in the preparation and characterization (especially structural characterization) of single crystals and ceramics. It should also be noted that the present/reported work is based on a ceramic sample. The reported structural data show that the addition of Co/Ti has distorted the basic structure of BiFeO_3_ from the perovskite rhombohedral symmetry to orthorhombic symmetry. Most of the peaks (except very few low intensity peaks) were indexed in the selected system. Hence, the new unit cell dimensions of the distorted symmetry have been obtained and presented in the published paper.^1^ To begin with, the structural analysis of the reported compound was tried in several crystal systems. The best agreement between the observed (experimental) and calculated inter-planar distances and diffraction angles of the new unit cell for the modified BiFeO_3_ was obtained in the orthorhombic system. The same indexing programme (POWDMULT software) was also utilized by a lot of research groups for other sets of compounds.^3–5^ The validity of the determined structure of the said compound is always tested for its consistency with the physical properties of the material, which has been done in the reported work.^1^ Moreover, there are several reports where splitting of peaks has been shown for a single phase system.^4^ For example, some higher angle reflections of tetragonal and orthorhombic systems have peak splitting (usually referred to as tetragonal/orthorhombic splitting).^6,7^

Most of the major (except very few low intensity peaks) reflections of the reported compound have been successfully indexed (with minimum standard deviation values) to a single phase; the proposed bi-phase/multiple phase for the reported compound may not be appropriate or acceptable, because the proposed compound needs different amounts of ingredients as compared to the reported one to maintain the stoichiometry. In conclusion, it is a well-known fact that the structural and electrical properties of ceramic specimens are greatly affected by a small variation in composition, and every composition of the system gives some new and interesting/important results. Thus, the proposed bi-phase for the reported compound has to be verified by detailed structural analysis with new experimental data. However, other possibilities of structural analysis using accurate data and advanced analysis techniques cannot be ruled out.

The chemical formula for the compound of sillenite type proposed by Paweł E. Tomaszewski is not clearly given and discussed in the comment. A quantitative comparison between the sillenite structure from the literature and the proposed structure is also needed to claim the same. Once again, it should be mentioned that the proposed compound Bi_25_FeO_39_ with Co/Ti needs different amounts of ingredients to those taken for the reported/presented compound, to maintain the stoichiometric ratio. The validity of the compound chemical reaction and chemical composition proposed by the commenter is still unknown. As the structural and physical properties are closely related to each other, detailed studies of these (crystal structure and physical characteristics) with accurate data are very much required. Finally, it can be concluded that the crystal symmetry of the published compound is quite different to that of the proposed one [by Paweł E. Tomaszewski]. Because of the difference between the compounds (presented and proposed), the structural data of the proposed compound cannot be compared with those of the published one.^1,8,9^ In view of the above scientific evidence/key points, the comments on the structural part of our published work have no merit and are unacceptable.

## Conflicts of interest

There are no conflicts to declare.

## Supplementary Material
